# Supplementary material for: Phenology, Seasonal Abundance, and Host-Plant Association of Spittlebugs (Hemiptera: Aphrophoridae) in Vineyards of Northwestern Italy
Source: Insects. 2021 Nov 9;12(11):1012. doi: 10.3390/insects12111012 (PMC8624594; doi:10.3390/insects12111012)
Supplement: Supplementary file 1 [file insects-12-01012-s001.zip › insects-1429099-supplementary.pdf]

Table S1. Dates of first, last and peak occurrences of the five nymphal instars and adults spittlebugs (peak dates = max no. of nymphs sampled)

		Philaenus spumarius			*Neophilaenus campestris*			*Aphrophora alni*		
		first	peak	last	first	peak	last	first	peak	last
2016	1st	23-Mar	23-Mar	23-Mar	—	—	—	—	—	—
	2nd	23-Mar	23-Mar	20-Apr	23-Mar	31-Mar	27-Apr	—	—	—
	3rd	23-Mar	12-Apr	05-May	23-Mar	12-Apr	05-May	—	—	—
	4th	31-Mar	12-Apr	05-May	12-Apr	27-Apr	05-May	27-Apr	27-Apr	05-May
	5th	12-Apr	05-May	05-May	12-Apr	27-Apr	05-May	27-Apr	05-May	05-May
	Ad	27-Apr	27-Sept	16-Dec	27-Apr	24-May	16-Dec	24.May	01-Sept	17-Oct
2017	1st	17-Mar	17-Mar	12-Apr	17-Mar	17-Mar	12-Apr	27-Mar	27-Mar	27-Mar
	2nd	17-Mar	27-Mar	02-May	17-Mar	12-Apr	12-Apr	27-Mar	12-Apr	02-May
	3rd	27-Mar	12-Apr	02-May	12-Apr	12-Apr	12-Apr	12-Apr	02-May	02-May
	4th	12-Apr	02-May	10-May	12-Apr	12-Apr	10-May	02-May	02-May	02-May
	5th	12-Apr	10-May	10-May	02-May	02-May	02-May	10-May	10-May	22-May
	Ad	02-May	30-May	22-Nov	10-May	19-Oct	22-Nov	22-May	30-May	19-Oct

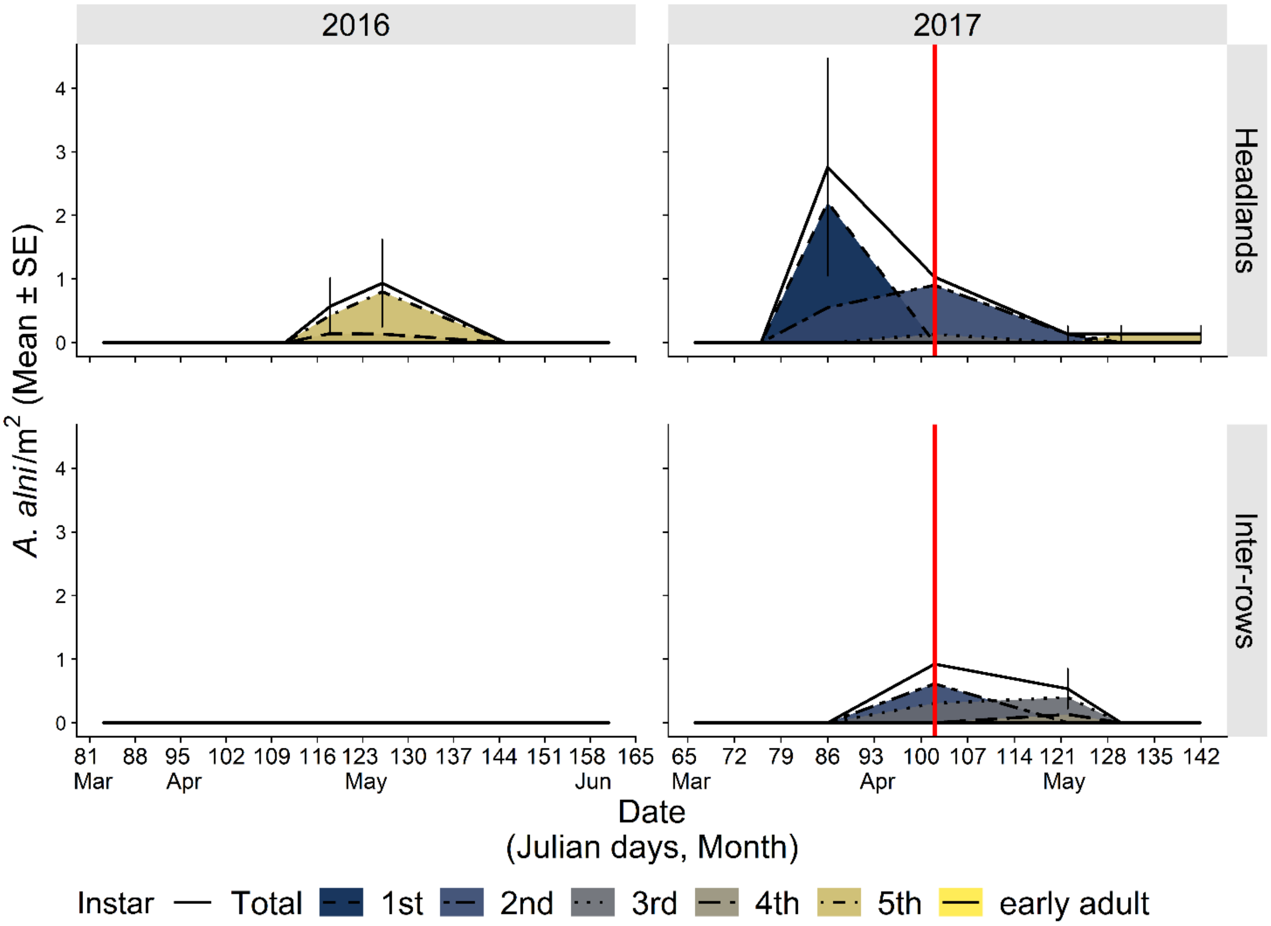

Figure S2. Life stage structure of the preimaginal populations of *Aphrophora alni* in vineyard inter-rows and headlands. The area curves of each nymphal instar, the total nymphs, and the newly emerged adults are based on the mean densities, as estimated by quadrat sampling on each sampling date. Red vertical lines represent the date of mowing treatment.

Table S3. Absolute (number) and relative (percentage) distributions of *Philaenus spumarius* nymphs according to plant genera on which they were found in an organic vineyard in Asti (North Western Italy) during 2016-2017 surveys. Plant genera are arranged in descending order of *Philaenus spumarius* nymphs observed on them.

Host plant taxon		No. nymphs sampled			% on total nymphs
**Genus**	**Family**	**2016**	**2017**	**Total**	
Plantago	Plantaginaceae	541	429	970	15.209
Cerastium	Caryophyllaceae	333	354	687	10.771
Trifolium	Fabaceae	474	137	611	9.580
Taraxacum	Asteraceae	220	199	419	6.569
Rumex	Polygonaceae	270	21	291	4.563
Medicago	Fabaceae	274	14	288	4.516
Erigeron	Asteraceae	175	86	261	4.092
Veronica	Plantaginaceae	130	130	260	4.077
Convolvulus	Convolvulaceae	245	9	254	3.982
Hypochaeris	Asteraceae	121	122	243	3.810
Picris	Asteraceae	219	15	234	3.669
Geranium	Geraniaceae	132	66	198	3.104
Poa	Poaceae	174	—	174	2.728
Crepis	Asteraceae	74	68	142	2.226
Sonchus	Asteraceae	121	—	121	1.897
Lotus	Fabaceae	39	64	103	1.615
Bellis	Asteraceae	43	42	85	1.333
Bromus	Poaceae	73	—	73	1.145
Avena	Poaceae	68	1	69	1.082
Salvia	Lamiaceae	51	11	62	0.972
Solidago	Asteraceae	52	7	59	0.925
Poaceae spp.	Poaceae	13	42	55	0.862
Asteraceae spp.	Asteraceae	12	33	45	0.706
Artemisia	Asteraceae	—	42	42	0.659
Stellaria	Caryophyllaceae	1	40	41	0.643
Galium	Rubiaceae	27	10	37	0.580
Vicia	Fabaceae	35	—	35	0.549
Potentilla	Rosaceae	18	17	35	0.549
Erodium	Geraniaceae	29	3	32	0.502
Rapistrum	Brassicaceae	—	31	31	0.486
Ranunculus	Ranunculuceae	31	—	31	0.486
Daucus	Apiaceae	27	1	28	0.439
Centaurea	Asteraceae	26	—	26	0.408
Fabaceae spp.	Fabaceae	26	—	26	0.408
Calepina	Brassicaceae	25	—	25	0.392
Myosotis	Boraginaceae	9	15	24	0.376

Caryophyllaceae spp.	Caryophyllaceae	—	24	24	0.376
Sanguisorba	Rosaceae	5	17	22	0.345
Cirsium	Asteraceae	17	—	17	0.267
Papaveraceae	Papaveraceae	—	14	14	0.220
Euphorbia	Euphorbiaceae	—	13	13	0.204
Cichorium	Asteraceae	—	12	12	0.188
Leucanthemum	Asteraceae	10	1	11	0.172
Brassicaceae	Brassicaceae		10	10	0.157
Sorghum	Poaceae	10	—	10	0.157
Hieracium	Asteraceae	6	1	7	0.110
Lactuca	Asteraceae	7	—	7	0.110
Mentha	Lamiaceae	6	—	6	0.094
Ranunculus	Ranunculaceae		6	6	0.094
Achillea	Asteraceae	2	3	5	0.078
Ornithogalum	Asparagaceae	4		4	0.063
Thesium	Thesiaceae	—	4	4	0.063
Muscari	Asparagaceae	3	—	3	0.047
Ambrosia	Asteraceae	3	—	3	0.047
Senecio	Asteraceae	—	3	3	0.047
Capsella	Brassicaceae	—	3	3	0.047
Liliaceae	Liliaceae	—	3	3	0.047
Leontodon	Asteraceae	1	1	2	0.031
Echium	Boraginaceae	2	—	2	0.031
Coronilla	Fabaceae	1	1	2	0.031
Hordeum	Poaceae	2	—	2	0.031
Lolium	Poaceae	2	—	2	0.031
Calendula	Asteraceae	1	—	1	0.016
Dianthus	Caryophyllaceae	1	—	1	0.016
Convolvulus	Convulvulaceae	1	—	1	0.016
Euphorbiaceae spp.	Euphorbiaceae	1	—	1	0.016
Lathyrus	Fabaceae	1	—	1	0.016
Carex	Poaceae	1	—	1	0.016
Alchemilla	Rosaceae	1	—	1	0.016
Fragaria	Rosaceae	1	—	1	0.016
Populus	Salicaceae	1	—	1	0.016
Solanum	Solanaceae	—	1	1	0.016
Not on plant		20	1	20	0.329
Unknown plant taxa		11	14	34	0.547

Table S4. Output of the generalized linear mixed models (GLMM) for the effects height of herbaceous cover (*height_herb_cover*), percentage of herbaceous cover (*%_herb_cover*), and vineyard zone on density of *Philaenus spumarius* nymphs (#/m^2) during peak period of 2016 survey (2016-03-20 – 2016-05-10).

Effects	Beta	95% CI[1]	p-value
Fixed			
**Intercept**	5.7	5.5, 6.0	**<0.001**
**height_herb_cover**	0.16	0.04, 0.28	**0.011**
**%_herb_cover**	0.06	-0.03, 0.16	0.18
**Zone**			0.057
Inter-rows	—	—	
Headlands	0.18	-0.01, 0.37	
Random			
Date SD_(Intercept)	0.19		

[1]CI = Confidence Interval

Table S5. Output of the linear mixed models (LMM) for the effects height of herbaceous cover (*height_herb_cover*), percentage of herbaceous cover (*%_herb_cover*), and vineyard zone on density of *Neophilaenus campestris* nymphs (#/m^2) during peak period of 2016 survey (2016-03-20 – 2016-05-10).

Effects	Beta	95% CI[1]	p-value
Fixed			
**Intercept**	5.0	4.6, 5.4	**<0.001**
**height_herb_cover**	-0.16	-0.40, 0.08	0.18
**%_herb_cover**	0.05	-0.21, 0.31	0.69
**Zone**			**<0.001**
Inter-rows	—	—	
Headlands	-0.67	-1.0, -0.31	
**height_herb_cover * %_herb_cover**	0.10	-0.09, 0.28	0.30
**height_herb_cover * Zone**			**<0.001**
*height_herb_cover * Headlands*	-0.71	-1.1, -0.32	
**%_herb_cover * Zone**			**0.018**
*%_herb_cover * Headlands*	0.44	0.08, 0.80	
Random			
Date SD (Intercept)	0.35		
Residual SD Observation	1.1		

[1]CI = Confidence Interval

ù

Table S6. Output of the binomial generalized linear mixed models (GLMM) for the effects Season, Vegetation component, and Year on sex ratio of *P. spumarius* adults.

Effects	log(OR)[1]	95% CI[1]	p-value
**(Intercept)**	-0.39	-0.80, 0.02	0.065
**Season**			**<0.001**
Apr-Jun	—	—	
Jul-Sept	-0.42	-1.0, 0.12	
Oct-Dec	-0.60	-2.1, 0.86	
**Vegetation_component**			0.61
Grapevine	—	—	
Herbaceous	0.09	-0.37, 0.54	
Shrubs-Trees	0.16	-0.28, 0.60	
**Year**			**0.010**
2016	—	—	
2017	0.09	-0.46, 0.65	
**Season * Vegetation_component**			0.80
*Jul-Sept * Herbaceous*	0.04	-0.52, 0.60	
*Oct-Dec * Herbaceous*	-0.30	-1.7, 1.1	
*Jul-Sept * Shrubs-Trees*	0.21	-0.35, 0.76	
*Oct-Dec * Shrubs-Trees*	0.47	-1.8, 2.8	
**Season * Year**			0.19
*Jul-Sept * 2017*	0.38	-0.04, 0.80	
*Oct-Dec * 2017*	0.13	-0.51, 0.77	
**Vegetation_component * Year**			0.077
*Herbaceous * 2017*	0.10	-0.46, 0.65	
*Shrubs-Trees * 2017*	-0.32	-0.89, 0.25	
**Date SD (Intercept)**	0.14		

[1]OR = Odds Ratio, CI = Confidence Interval

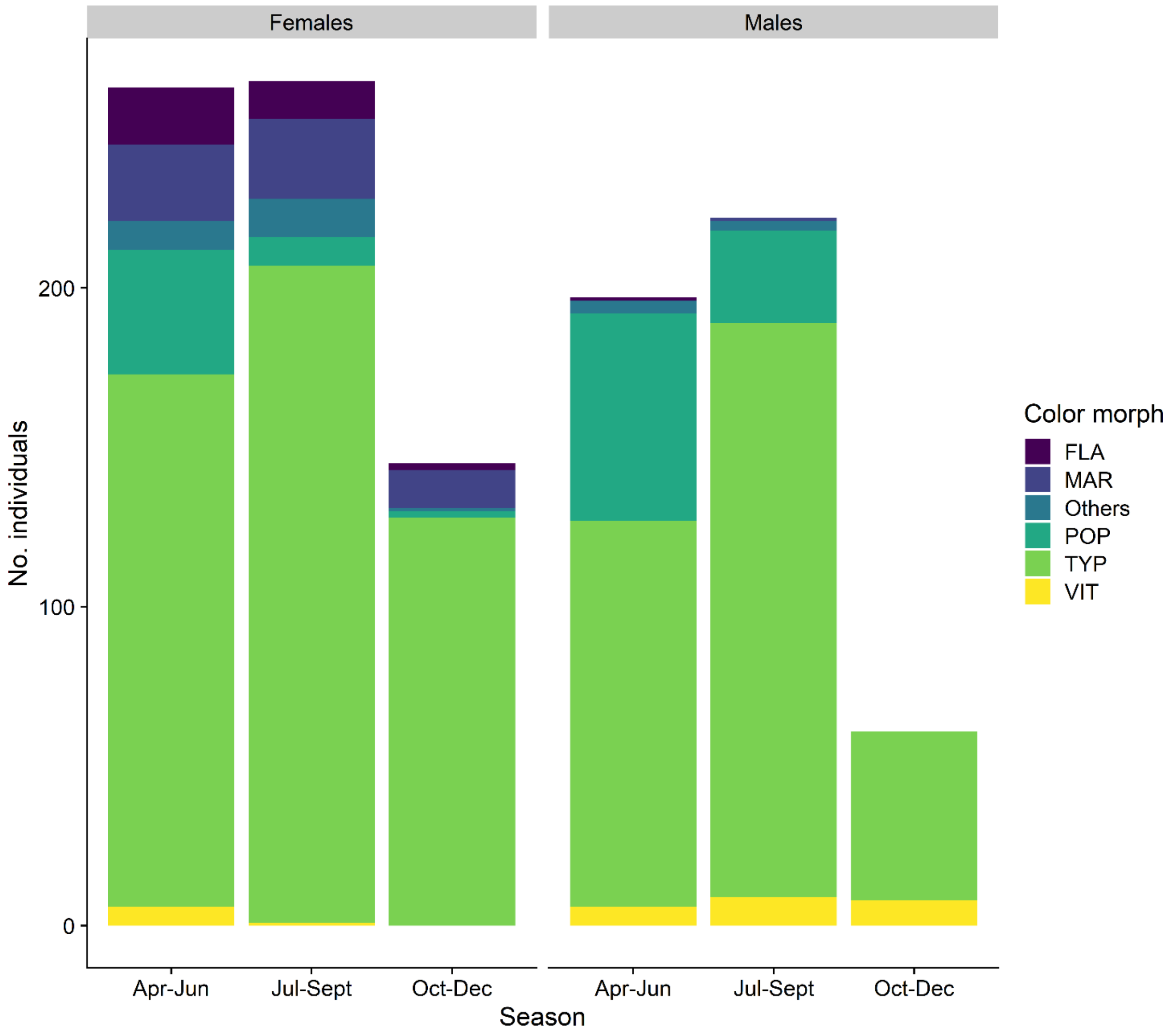

Figure S7. Prevalence of principal color morphs of *Philaenus spumarius* adults in the Asti vineyard during 2016-2017 surveys. Data aggregated per sex and sampling season.

Table S8. Output of the negative binomial generalized linear models (GLM) for the effects Season, Sex, and Vegetation component on colour morphs frequency among *P. spumarius* adults during 2017 survey.

Effects	Beta	95% CI[1]	p-value
**(Intercept)**	0.14	-1.8, 2.8	0.90
**Colour_morph * Season**			0.88
*MAR * Jul-Sept*	0.76	-2.5, 4.2	
*Others * Jul-Sept*	1.0	-2.0, 4.1	
*POP * Jul-Sept*	-0.15	-2.9, 2.8	
*TYP * Jul-Sept*	1.1	-1.7, 4.0	
*VIT * Jul-Sept*	0.34	-2.8, 3.7	
*MAR * Oct-Dec*	1.4	-2.6, 5.7	
*Others * Oct-Dec*	0.35	-3.9, 4.7	
*POP * Oct-Dec*	-1.1	-4.8, 3.0	
*TYP * Oct-Dec*	1.6	-1.8, 5.6	
*VIT * Oct-Dec*	1.5	-2.3, 5.7	
**Colour_morph * Sex**			**<0.001**
*MAR * Male*	-0.50	-4.9, 4.0	
*Others * Male*	3.2	-0.01, 7.3	
*POP * Male*	4.3	1.3, 8.4	
*TYP * Male*	3.5	0.62, 7.5	
*VIT * Male*	5.7	2.4, 9.9	
**Colour_morph * Vegetation_component**			>0.99
*MAR * Herbaceous*	1.0	-3.2, 5.2	
*Others * Herbaceous*	-0.54	-4.6, 3.3	
*POP * Herbaceous*	-0.65	-4.5, 2.7	
*TYP * Herbaceous*	0.30	-3.4, 3.5	
*VIT * Herbaceous*	1.2	-3.0, 5.4	
*MAR * Shrubs-Trees*	0.71	-3.0, 4.5	
*Others * Shrubs-Trees*	0.28	-3.3, 3.9	
*POP * Shrubs-Trees*	-0.43	-3.7, 2.8	
*TYP * Shrubs-Trees*	-0.05	-3.2, 3.0	
*VIT * Shrubs-Trees*	0.67	-3.1, 4.9	

[1]CI = Confidence Interval
